# Supplementary material for: Analysis of PPARγ Signaling Activity in Psoriasis
Source: Int J Mol Sci. 2021 Aug 10;22(16):8603. doi: 10.3390/ijms22168603 (PMC8395241; doi:10.3390/ijms22168603)
Supplement: Supplementary file 1 [file ijms-22-08603-s001.zip › Supplemental materials_Analysis of PPARg signaling activity in psoriasis/Pathway models/Models images and html files/Model of PPARG related pathways in psoriasis (short version)/9530.html]

PTK2


# Protein PTK2

|  |  |
| --- | --- |
| URN | urn:agi-llid:5747 |
| Total Entities | 1 |
| Connectivity | 8007 |
| Name | PTK2 |
| Description | protein tyrosine kinase 2 |
| Notes | This gene encodes a cytoplasmic protein tyrosine kinase which is found concentrated in the focal adhesions that form between cells growing in the presence of extracellular matrix constituents. The encoded protein is a member of the FAK subfamily of protein tyrosine kinases but lacks significant sequence similarity to kinases from other subfamilies. Activation of this gene may be an important early step in cell growth and intracellular signal transduction pathways triggered in response to certain neural peptides or to cell interactions with the extracellular matrix. Several transcript variants encoding different isoforms have been found for this gene. [provided by RefSeq, Jun 2017] |
| Primary Cell Localization | Cytoplasm |
| Class | Protein kinase |
| ObjectType | Protein |

---

|  |  |
| --- | --- |
| ChildConcepts | FRNK |

---

|  |  |
| --- | --- |
| Pathway | Thyroid Hormones Common non-Genomic Effects in Hyperthyroidism |
|  | CXC Chemokine Receptor Signaling |
|  | CC Chemokine Receptor Signaling |
|  | Platelet Activation via Adhesion Molecules |
|  | Eat me Signal: Apoptotic Cell Induces Phagocytosis |
|  | Neutrophil Activation via Adherence on Endothelial Cells |
|  | Vascular Endothelial Cell Permeability Activation |
|  | CHRNA7 -> NOS1 Production |
|  | EPHB -> NMDA Receptor Activation |
|  | G0/G1 Phase Transition |
|  | Focal Junction Assembly |
|  | Proteins Involved in Dilated Cardiomyopathy |
|  | Proteins Involved in Arterial Hypertension |
|  | Proteins Involved in Myocardial Ischemia |
|  | Proteins with Altered Expression in Melanoma |
|  | Melanoma |
|  | Apoptosis Block in Melanoma Cell |
|  | Dedifferentiation and Metastatic Progression in Melanoma |
|  | Proteins Involved in Melanoma |
|  | Neutrophil and Macrophage Function in Systemic Lupus Erythematosus |
|  | Proteins Involved in Diabetic Nephropathy |
|  | Proliferative Diabetic Retinopathy |
|  | RET1 Mutation in Hyperparathyroidism |
|  | Cardiovascular Effects in Hyperthyroidism |
|  | Myocyte Dysfunction in Hypoparathyroidism |
|  | Metastatic Colorectal Cancer |
|  | OipA Signaling in Helicobacter Pylori Infection |
|  | CagA Phosphorylation Independent Signaling |
|  | CagA Phosphorylation Dependent Signaling |
|  | Proteins Involved in Hepatocellular Carcinoma |
|  | Proteins Involved in Pancreatic Cancer |
|  | Goblet-Cell Exocytosis in Asthma |
|  | Neutrophil Degranulation via FPR1/IL8 |
|  | Synovial Fibroblast Proliferation in Rheumatoid Arthritis |
|  | WHIM Syndrome |
|  | Macrophage Survival through CCR5 and CXCR4 Mediated Signaling |
|  | Cowden Syndrome |
|  | Ca2+ Overload in Muscular Dystrophies |
|  | Muscular Dystrophies |
|  | Dystrophin Glycoprotein Complex Signaling in Muscular Dystrophies |
|  | Glioma Invasion Signaling |
|  | Proteins Involved in Glioma |
|  | Ependymoma |
|  | Proteins Involved in Neuroblastoma |
|  | Proteins Involved in Breast Cancer Related to ESR1 Signaling Pathway |
|  | Proteins Involved in Breast Cancer Related to ERBB2/VEGFR/Akt Signaling Pathway |
|  | Proteins Involved in Breast Cancer Related to IGF1R/Akt Signaling Pathway |
|  | Proteins Involved in Endometriosis |
|  | Angiogenesis in Endometriosis |
|  | HPV E5 Regulates EDNRA and EGFR Signaling Pathway in Keratinocytes |
|  | Prostate Cancer |
|  | Proteins Involved in Prostate Cancer |
|  | Proteins Involved in Cataract |
|  | VEGFA in Neovascular Glaucoma |
|  | CD157/ITGB2 Signaling in Myeloid Cell |
|  | Acetylcholine Expression Targets |
|  | Aryl Hydrocarbon Receptor Genomic and non-Genomic Signaling |
|  | Elevated Receptors -> Expression Targets in Bone |
|  | CHRNA7 -> CREB Signaling |
|  | NCAM1 Expression Targets |
|  | FGF2 -> AP-1/CREB/CREBBP/ELK/SRF/MYC Expression Targets |
|  | Receptors and Adaptor Proteins Activated in Cancer |
|  | Proteins with Altered Expression in Cancer-Associated Sustaining of Proliferative Signaling |
|  | G0/G1 Cell Cycle Phase Transition Activation in Cancer |
|  | HIF1A in Vasculogenic Mimicry of Cancer |
|  | ANGPT/TEK Stimulates Endothelial Cell Migration in Cancer |
|  | VEGF Independent Angiogenesis in Cancer |
|  | Integrins in Cancer Cell Motility, Invasion and Survival |
|  | Proteins with Altered Expression in Cancer Metastases |
|  | Epithelial to Mesenchymal Transition in Cancer: Overview |
|  | BSG (CD147) in Cancer Cells Motility, Invasion and Survival |
|  | CDH2 Activation Promotes Cancer Cell Migration and Survival |
|  | Lipogenesis/Lipolysis Activation in Cancer Cells and Cancer-Associated Adipocytes |
|  | NCAM1 -> CREB/ELK/SRF/MYC Signaling |
|  | FibronectinR -> CTNNB Signaling |
|  | FibronectinR -> NF-kB Signaling |
|  | FibronectinR -> AP-1/ELK/SRF/SREBF Signaling |
|  | Fibronectin Expression Targets |
|  | EphrinB -> Cytoskeleton Signaling |
|  | EphrinB -> MAPK/JUN/FOS Signaling |
|  | FOXO1 Signaling |
|  | MERTK Signaling |
|  | PI3K/RAC1 Signaling |
|  | VEGF Signaling |
|  | EGFR Signaling |
|  | ERK5/MAPK7 Signaling |
|  | ERK/MAPK Canonical Signaling |
|  | VEGFC -> CTNNB Expression Target |
|  | VEGFA -> STAT Expression Targets |
|  | VEGFA -> CTNNB/CTNND Expression Targets |
|  | VEGFR -> CTNNB Signaling |
|  | VEGFR -> STAT Signaling |
|  | Model of PPARG signaling in psoriasis |
|  | Model of PPARG related pathways in psoriasis (short version) |
|  | Possible Interplay between SOX11, CCND1 and EZH2 in Mantle Cell Lymphoma |
|  | 3\_Enhanced angiogenesis in endometriosis |
|  | 2\_Pathogens stimulate mucins expression in the middle ear |
|  | 3\_1\_Mucus accumulation in asthma |
|  | 4\_2\_Airway smooth muscle cells proliferation and airway tissue remodeling |
|  | 4\_Mucin hyperproduction in goblet and mucous cells in COPD |
|  | 2\_1\_General pathways of thyroid hormone signaling |
|  | Model of PPARG signaling in psoriais (tested) |

---

|  |  |
| --- | --- |
| Group | Proteins Involved in Cowden Syndrome |

---

|  |  |
| --- | --- |
| MedScan ID | 5747 |

---

|  |  |
| --- | --- |
| LocusLink ID | 5747 |
|  | 14083 |
|  | 25614 |

---

|  |  |
| --- | --- |
| Alias | PTK2 protein tyrosine kinase |
|  | focal adhesion kinase p125 |
|  | PTK-2 |
|  | Focal adhesion kinase I |
|  | FAK |
|  | FADK |
|  | p125FAK |
|  | PTK2 |
|  | focal adhesion kinase |
|  | FAK I |
|  | mKIAA4203 |
|  | PTK2 protein tyrosine kinase 2 |
|  | PPP1R71 |
|  | FADK I |
|  | focal ashension kinase 1 |
|  | FADK 1 |
|  | pp125FAK |
|  | protein tyrosine kinase 2 |
|  | Focal adhesion kinase 1 |
|  | protein phosphatase 1, regulatory subunit 71 |
|  | FAK1 |
|  | focal adhesion kinase isoform FAK-Del33 |
|  | PTK2 protein tyrosine kinase II |
|  | KIAA4203 |
|  | FRNK |
|  | FAK-related non-kinase polypeptide |
|  | focal adhesion kinase-related nonkinase |
|  | protein phosphatase 1 regulatory subunit 71 |
|  | protein-tyrosine kinase 2 |
|  | FAK-related non kinase polypeptide |
|  | FAK-related nonkinase polypeptide |

---

|  |  |
| --- | --- |
| EC Number | 2.7.10.2 |

---

|  |  |
| --- | --- |
| GO ID | 0005524 |
|  | 0008432 |
|  | 0042169 |
|  | 0003779 |
|  | 0004715 |
|  | 0004672 |
|  | 0019901 |
|  | 0019903 |
|  | 0004713 |
|  | 0038096 |
|  | 0000165 |
|  | 0001525 |
|  | 0007411 |
|  | 0030154 |
|  | 0048870 |
|  | 0021955 |
|  | 0043542 |
|  | 0048013 |
|  | 0007173 |
|  | 0030010 |
|  | 0030198 |
|  | 0060396 |
|  | 0003007 |
|  | 0007229 |
|  | 0000226 |
|  | 2000811 |
|  | 0043066 |
|  | 0010507 |
|  | 0050771 |
|  | 0022408 |
|  | 0046621 |
|  | 0051964 |
|  | 0038007 |
|  | 0001764 |
|  | 0007097 |
|  | 0038083 |
|  | 0018108 |
|  | 0001890 |
|  | 0010613 |
|  | 0030335 |
|  | 0008284 |
|  | 0043552 |
|  | 0014068 |
|  | 0051897 |
|  | 0045860 |
|  | 0001934 |
|  | 2000060 |
|  | 0046777 |
|  | 0043087 |
|  | 0030155 |
|  | 0033628 |
|  | 0042127 |
|  | 0008360 |
|  | 0051493 |
|  | 0010594 |
|  | 0010632 |
|  | 0051893 |
|  | 0045667 |
|  | 0001932 |
|  | 1900024 |
|  | 0007172 |
|  | 0007179 |
|  | 0048010 |
|  | 0001570 |
|  | 0005912 |
|  | 0016324 |
|  | 0005938 |
|  | 0005737 |
|  | 0005856 |
|  | 0005829 |
|  | 0043197 |
|  | 0005925 |
|  | 0030027 |
|  | 0005815 |
|  | 0005634 |
|  | 0005886 |
|  | 0001725 |
|  | 0005178 |
|  | 0016301 |
|  | 0000166 |
|  | 0019902 |
|  | 0043548 |
|  | 0044877 |
|  | 0016740 |
|  | 0031625 |
|  | 0007254 |
|  | 0001568 |
|  | 0030644 |
|  | 0071560 |
|  | 0007275 |
|  | 0030336 |
|  | 0016310 |
|  | 0097755 |
|  | 0045785 |
|  | 0030307 |
|  | 0060252 |
|  | 0050766 |
|  | 0014911 |
|  | 0048661 |
|  | 0050806 |
|  | 0006468 |
|  | 0016323 |
|  | 0030054 |
|  | 0098978 |
|  | 0014704 |
|  | 0016020 |
|  | 0016604 |
|  | 0005730 |
|  | 0098794 |
|  | 0042383 |
|  | 0045444 |
|  | 0046685 |
|  | 0042493 |
|  | 0032355 |
|  | 0009749 |
|  | 0009612 |
|  | 0014070 |
|  | 0010033 |
|  | 0010243 |
|  | 0048471 |
|  | 0045087 |
|  | 0031234 |
|  | 0005102 |
|  | 0032319 |
|  | 0005515 |
|  | 0009790 |
|  | 0007010 |
|  | 0004871 |
|  | 0040023 |
|  | 0007596 |
|  | 0032403 |
|  | 0006915 |
|  | 0043547 |
|  | 0007165 |
|  | 0007169 |
|  | 0006921 |
|  | 0006928 |
|  | 0030168 |
|  | 0016772 |
|  | 0005088 |
|  | 0045909 |
|  | 0007155 |

---

|  |  |
| --- | --- |
| KEGG ID | hsa:5747 |
|  | mmu:14083 |
|  | rno:25614 |

---

|  |  |
| --- | --- |
| Organism | Homo sapiens {Organism urn:agi-taxid:9606} |
|  | Mus musculus {Organism urn:agi-taxid:10090} |
|  | Rattus norvegicus {Organism urn:agi-taxid:10116} |
|  | Homo sapiens |
|  | Mus musculus |
|  | Rattus norvegicus |

---

|  |  |
| --- | --- |
| Mouse chromosome position | 15 33.94 cM |
|  | 15 42.0 cM |

---

|  |  |
| --- | --- |
| OMIM ID | 600758 |

---

|  |  |
| --- | --- |
| Rat chromosome position | 7q34 |

---

|  |  |
| --- | --- |
| Hugo ID | 9611 |
|  | HGNC:9611 |

---

|  |  |
| --- | --- |
| Human chromosome position | 8q24.3 |

---

|  |  |
| --- | --- |
| Swiss-Prot Accession | Q05397 |
|  | Q59GM6 |
|  | Q658W2 |
|  | Q59GN8 |
|  | Q05397.2 |
|  | P34152 |
|  | K7Q751 |
|  | P34152.4 |
|  | O35346 |
|  | O35346.1 |
|  | B4E2N6 |
|  | F5H4S4 |
|  | J3QT16 |
|  | Q14291 |
|  | Q8IYN9 |
|  | Q9UD85 |
|  | O08578 |
|  | Q5DTH7 |
|  | Q8C513 |
|  | Q8CFH7 |
|  | Q8CHM2 |
|  | Q8K2S0 |
|  | Q9DAW3 |
|  | Q62900 |
|  | P34152.3 |
|  | R9PXT6 |
|  | E7ESA6 |

---

|  |  |
| --- | --- |
| PIR ID | I53012 |
|  | PC1225 |
|  | A46166 |

---

|  |  |
| --- | --- |
| GenBank ID | NC\_000008 |
|  | NR\_148039 |
|  | NR\_148038 |
|  | NR\_148036 |
|  | NR\_148037 |
|  | NM\_001352699 |
|  | NP\_001339628 |
|  | NM\_001352746 |
|  | NP\_001339675 |
|  | NM\_001352742 |
|  | NP\_001339671 |
|  | NM\_001352745 |
|  | NP\_001339674 |
|  | NM\_001352744 |
|  | NP\_001339673 |
|  | NM\_001352741 |
|  | NP\_001339670 |
|  | NM\_001352738 |
|  | NP\_001339667 |
|  | NM\_001316342 |
|  | NP\_001303271 |
|  | NM\_001352733 |
|  | NP\_001339662 |
|  | NM\_001352734 |
|  | NP\_001339663 |
|  | NM\_001352732 |
|  | NP\_001339661 |
|  | NM\_001352730 |
|  | NP\_001339659 |
|  | NM\_001352728 |
|  | NP\_001339657 |
|  | NM\_001352729 |
|  | NP\_001339658 |
|  | NM\_001352711 |
|  | NP\_001339640 |
|  | NM\_001352694 |
|  | NP\_001339623 |
|  | NM\_001352720 |
|  | NP\_001339649 |
|  | NM\_001352713 |
|  | NP\_001339642 |
|  | NM\_001352715 |
|  | NP\_001339644 |
|  | NM\_001352727 |
|  | NP\_001339656 |
|  | NM\_001352721 |
|  | NP\_001339650 |
|  | NM\_001352706 |
|  | NP\_001339635 |
|  | NM\_001352747 |
|  | NP\_001339676 |
|  | NM\_001352700 |
|  | NP\_001339629 |
|  | NM\_001352736 |
|  | NP\_001339665 |
|  | NM\_001352740 |
|  | NP\_001339669 |
|  | NM\_001352743 |
|  | NP\_001339672 |
|  | NM\_001352696 |
|  | NP\_001339625 |
|  | NM\_001352731 |
|  | NP\_001339660 |
|  | NM\_001352726 |
|  | NP\_001339655 |
|  | NM\_001352716 |
|  | NP\_001339645 |
|  | NM\_153831 |
|  | NP\_722560 |
|  | NM\_001352718 |
|  | NP\_001339647 |
|  | NM\_001199649 |
|  | NP\_001186578 |
|  | NM\_001352714 |
|  | NP\_001339643 |
|  | NM\_001352717 |
|  | NP\_001339646 |
|  | NM\_001352723 |
|  | NP\_001339652 |
|  | NM\_001352724 |
|  | NP\_001339653 |
|  | NM\_001352709 |
|  | NP\_001339638 |
|  | NM\_001352695 |
|  | NP\_001339624 |
|  | NM\_001352704 |
|  | NP\_001339633 |
|  | NM\_001352702 |
|  | NP\_001339631 |
|  | NM\_001352701 |
|  | NP\_001339630 |
|  | NM\_001352735 |
|  | NP\_001339664 |
|  | NM\_001352719 |
|  | NP\_001339648 |
|  | NM\_001352722 |
|  | NP\_001339651 |
|  | NM\_001352708 |
|  | NP\_001339637 |
|  | NM\_001352725 |
|  | NP\_001339654 |
|  | NM\_001352739 |
|  | NP\_001339668 |
|  | NM\_001352707 |
|  | NP\_001339636 |
|  | NM\_001352703 |
|  | NP\_001339632 |
|  | NM\_001352712 |
|  | NP\_001339641 |
|  | NM\_001352737 |
|  | NP\_001339666 |
|  | NM\_005607 |
|  | NP\_005598 |
|  | NM\_001352697 |
|  | NP\_001339626 |
|  | NM\_001352705 |
|  | NP\_001339634 |
|  | NM\_001352698 |
|  | NP\_001339627 |
|  | NM\_001352710 |
|  | NP\_001339639 |
|  | NM\_001352752 |
|  | NP\_001339681 |
|  | NM\_001352751 |
|  | NP\_001339680 |
|  | NM\_001352750 |
|  | NP\_001339679 |
|  | NM\_001352749 |
|  | NP\_001339678 |
|  | XM\_024447210 |
|  | XP\_024302978 |
|  | XM\_024447207 |
|  | XP\_024302975 |
|  | XM\_024447206 |
|  | XP\_024302974 |
|  | XM\_024447205 |
|  | XP\_024302973 |
|  | XM\_024447203 |
|  | XP\_024302971 |
|  | XM\_017013666 |
|  | XP\_016869155 |
|  | XM\_017013669 |
|  | XP\_016869158 |
|  | XM\_024447202 |
|  | XP\_024302970 |
|  | XM\_017013656 |
|  | XP\_016869145 |
|  | XM\_017013688 |
|  | XP\_016869177 |
|  | XM\_017013684 |
|  | XP\_016869173 |
|  | XM\_024447211 |
|  | XP\_024302979 |
|  | XM\_024447209 |
|  | XP\_024302977 |
|  | XM\_024447208 |
|  | XP\_024302976 |
|  | XM\_024447200 |
|  | XP\_024302968 |
|  | XM\_024447199 |
|  | XP\_024302967 |
|  | XM\_017013654 |
|  | XP\_016869143 |
|  | XM\_017013653 |
|  | XP\_016869142 |
|  | XM\_024447212 |
|  | XP\_024302980 |
|  | XM\_017013664 |
|  | XP\_016869153 |
|  | XM\_024447204 |
|  | XP\_024302972 |
|  | XM\_024447201 |
|  | XP\_024302969 |
|  | XM\_017013657 |
|  | XP\_016869146 |
|  | NM\_001352748 |
|  | NP\_001339677 |
|  | NG\_029467 |
|  | AC067931 |
|  | AC100860 |
|  | AC105009 |
|  | AC105235 |
|  | AY323812 |
|  | CH471060 |
|  | EAW92210 |
|  | EAW92211 |
|  | EAW92212 |
|  | EAW92213 |
|  | EAW92214 |
|  | KF458878 |
|  | KF458882 |
|  | AB209071 |
|  | BAD92308 |
|  | AB209083 |
|  | BAD92320 |
|  | AK055139 |
|  | AK094999 |
|  | BAC04470 |
|  | AK124810 |
|  | AK294873 |
|  | BAG57974 |
|  | AK301557 |
|  | BAG63053 |
|  | AK304356 |
|  | BAG65198 |
|  | AL832961 |
|  | CAH56296 |
|  | BC015361 |
|  | BC028733 |
|  | AAH28733 |
|  | BC035404 |
|  | AAH35404 |
|  | BC043202 |
|  | BF434494 |
|  | BY800117 |
|  | DA236314 |
|  | DA994616 |
|  | DB478006 |
|  | KF437463 |
|  | AHZ89389 |
|  | L13616 |
|  | AAA58469 |
|  | Q05397 |
|  | NC\_000081 |
|  | XM\_006520426 |
|  | XP\_006520489 |
|  | XM\_006520430 |
|  | XP\_006520493 |
|  | XM\_006520431 |
|  | XP\_006520494 |
|  | XR\_383981 |
|  | XM\_006520435 |
|  | XP\_006520498 |
|  | XM\_006520424 |
|  | XP\_006520487 |
|  | XM\_006520416 |
|  | XP\_006520479 |
|  | XM\_006520425 |
|  | XP\_006520488 |
|  | XM\_006520415 |
|  | XP\_006520478 |
|  | XM\_006520429 |
|  | XP\_006520492 |
|  | XM\_006520428 |
|  | XP\_006520491 |
|  | XM\_006520427 |
|  | XP\_006520490 |
|  | XM\_006520434 |
|  | XP\_006520497 |
|  | XM\_017316429 |
|  | XP\_017171918 |
|  | XM\_006520433 |
|  | XP\_006520496 |
|  | XM\_006520423 |
|  | XP\_006520486 |
|  | XM\_006520422 |
|  | XP\_006520485 |
|  | XM\_006520421 |
|  | XP\_006520484 |
|  | XM\_006520420 |
|  | XP\_006520483 |
|  | XM\_006520418 |
|  | XP\_006520481 |
|  | XM\_011245453 |
|  | XP\_011243755 |
|  | NM\_007982 |
|  | NP\_032008 |
|  | XM\_006520432 |
|  | XP\_006520495 |
|  | XM\_017316428 |
|  | XP\_017171917 |
|  | XM\_011245452 |
|  | XP\_011243754 |
|  | NM\_001130409 |
|  | NP\_001123881 |
|  | XR\_001781493 |
|  | NM\_001358045 |
|  | NM\_001358046 |
|  | AC121319 |
|  | AC132863 |
|  | AC161581 |
|  | AH005806 |
|  | AAB95262 |
|  | AAB95263 |
|  | CH466545 |
|  | EDL29415 |
|  | EDL29416 |
|  | EDL29417 |
|  | EDL29418 |
|  | U77074 |
|  | AAB51229 |
|  | U77075 |
|  | AAB51230 |
|  | U77076 |
|  | AAB51231 |
|  | AB011499 |
|  | BAC53890 |
|  | AB030035 |
|  | BAC53924 |
|  | AK005468 |
|  | BAB24058 |
|  | AK052252 |
|  | AK079821 |
|  | BAC37757 |
|  | AK135490 |
|  | AK220543 |
|  | BAD90317 |
|  | AY916078 |
|  | AAY16304 |
|  | BB816209 |
|  | BC030180 |
|  | BU512211 |
|  | BY297123 |
|  | CJ050166 |
|  | DQ478404 |
|  | ABF47284 |
|  | DQ478405 |
|  | ABF47285 |
|  | DQ478406 |
|  | ABF47286 |
|  | DQ478407 |
|  | ABF47287 |
|  | JN971016 |
|  | AFR23585 |
|  | M95408 |
|  | AAA37592 |
|  | U15088 |
|  | P34152 |
|  | NC\_005106 |
|  | XM\_017594678 |
|  | XP\_017450167 |
|  | NM\_013081 |
|  | NP\_037213 |
|  | AC\_000075 |
|  | AABR07058402 |
|  | AABR07058403 |
|  | AAHX01051533 |
|  | AAHX01051534 |
|  | AAHX01051535 |
|  | CH473950 |
|  | EDM16117 |
|  | EDM16118 |
|  | EDM16119 |
|  | AF020777 |
|  | AAB72203 |
|  | AI717382 |
|  | CA508631 |
|  | CA511845 |
|  | CB713440 |
|  | EV773243 |
|  | U43940 |
|  | AAA86278 |
|  | U43941 |
|  | AAA86279 |
|  | U43942 |
|  | AAA86280 |
|  | O35346 |
|  | XM\_011517184 |
|  | XM\_011517185 |
|  | ABW03595 |
|  | XM\_011517186 |
|  | XM\_011517187 |
|  | XM\_011517188 |
|  | XM\_011517189 |
|  | XM\_011517180 |
|  | XM\_011517181 |
|  | XM\_011517182 |
|  | XM\_011517183 |
|  | XP\_011515483 |
|  | XP\_011515482 |
|  | XP\_011515489 |
|  | XP\_011515488 |
|  | XP\_011515485 |
|  | XP\_011515484 |
|  | XP\_011515487 |
|  | XP\_011515486 |
|  | XP\_011515492 |
|  | XP\_011515491 |
|  | XP\_011515494 |
|  | XP\_011515493 |
|  | XP\_011515490 |
|  | XP\_011515499 |
|  | XP\_011515496 |
|  | XP\_011515495 |
|  | XP\_011515498 |
|  | XP\_011515497 |
|  | XP\_006716669 |
|  | XM\_008765546 |
|  | XP\_006716673 |
|  | XP\_006716672 |
|  | XP\_006716671 |
|  | AMYH02018889 |
|  | AMYH02018888 |
|  | XP\_006716670 |
|  | XM\_011517195 |
|  | XM\_011517196 |
|  | XM\_011517197 |
|  | XM\_011517198 |
|  | AMYH02018890 |
|  | AAHY01123319 |
|  | AMYH02018892 |
|  | AMYH02018891 |
|  | AMYH02018894 |
|  | AMYH02018893 |
|  | XM\_011517190 |
|  | AMYH02018895 |
|  | XM\_011517191 |
|  | XM\_011517192 |
|  | XM\_011517193 |
|  | XM\_011517194 |
|  | AAHY01123323 |
|  | AAHY01123321 |
|  | AAHY01123322 |
|  | AAHY01123320 |
|  | XP\_005251067 |
|  | XP\_005251066 |
|  | XP\_005251069 |
|  | XP\_005251068 |
|  | XP\_005251063 |
|  | XP\_005251062 |
|  | XP\_005251065 |
|  | XP\_005251064 |
|  | XP\_005251061 |
|  | XP\_005251060 |
|  | XP\_008763768 |
|  | ABBA01013677 |
|  | ABBA01013679 |
|  | ABBA01013678 |
|  | XP\_005251070 |
|  | XP\_005251071 |
|  | AF025650 |
|  | AF025651 |
|  | AF025652 |
|  | AF025648 |
|  | AF025649 |
|  | XP\_006241787 |
|  | XP\_006520482 |
|  | XP\_006520480 |
|  | XM\_006241725 |
|  | XM\_006520417 |
|  | XM\_006520419 |
|  | AC\_000037 |
|  | AABR06051811 |
|  | AABR06051812 |
|  | AABR06051810 |
|  | XM\_005251013 |
|  | XM\_005251012 |
|  | XM\_005251011 |
|  | XM\_005251010 |
|  | XM\_006716609 |
|  | XM\_006716608 |
|  | XM\_006716607 |
|  | XM\_006716606 |
|  | XM\_005251014 |
|  | NC\_018919 |
|  | XM\_005251009 |
|  | XM\_005251008 |
|  | XM\_005251007 |
|  | XM\_005251006 |
|  | XM\_005251005 |
|  | XM\_005251004 |
|  | XM\_005251003 |
|  | XM\_006716610 |
|  | XP\_011515500 |
|  | EU176794 |
|  | AC\_000140 |

---

|  |  |
| --- | --- |
| Swiss-Prot ID | FAK1\_HUMAN |
|  | FAK1\_MOUSE |
|  | FAK1\_RAT |

---

|  |  |
| --- | --- |
| Cell Localization | Cell junction |
|  | Focal adhesion |
|  | Cell membrane |
|  | Cytoplasm |
|  | Cell cortex |
|  | Cytoskeleton |
|  | Microtubule organizing center |
|  | Centrosome |
|  | Nucleus |
|  | Perinuclear region |
|  | Cytoplasm, cell cortex |
|  | Cytoplasm, cytoskeleton |
|  | Cytoplasm, perinuclear region |
|  | Cell junction, focal adhesion |
|  | Cytoplasm, cytoskeleton, microtubule organizing center, centrosome |

---

|  |  |
| --- | --- |
| Ensembl ID | ENSG00000169398 |
|  | ENSP00000341189.3 |
|  | ENST00000340930.7 |
|  | ENSP00000429474.1 |
|  | ENST00000521059.5 |
|  | ENSP00000428492.1 |
|  | ENST00000523539.5 |
|  | ENSP00000429170.1 |
|  | ENST00000519465.5 |
|  | ENSP00000403416.2 |
|  | ENST00000430260.6 |
|  | ENSP00000429911.1 |
|  | ENST00000522684.5 |
|  | ENST00000519993.5 |
|  | ENSP00000429082.1 |
|  | ENST00000517887.5 |
|  | ENSP00000429129.1 |
|  | ENST00000519419.5 |
|  | ENSMUSG00000022607 |
|  | ENSMUSP00000105663.2 |
|  | ENSMUST00000110036.10 |
|  | ENSMUSP00000154242.1 |
|  | ENSMUST00000226988.2 |
|  | ENSRNOG00000007916 |
|  | ENSRNOP00000011219.6 |
|  | ENSRNOT00000011219.6 |
|  | ENSRNOP00000075518.1 |
|  | ENSRNOT00000079599.1 |
|  | ENSMUSP00000105663 |
|  | ENSMUST00000110036 |
|  | ENSP00000341189 |
|  | ENSRNOP00000075518 |
|  | ENST00000522684 |
|  | ENSMUST00000170939 |
|  | ENSMUSP00000126764 |
|  | ENSP00000429911 |
|  | ENSRNOP00000011219 |
|  | ENSP00000429082 |
|  | ENSRNOT00000011219 |
|  | ENSRNOT00000079599 |
|  | ENST00000517887 |
|  | ENST00000340930 |

---

|  |  |
| --- | --- |
| MGI ID | MGI:95481 |
|  | 95481 |

---

|  |  |
| --- | --- |
| RGD ID | 3443 |

---

|  |  |
| --- | --- |
| Unigene ID | Rn.2809 |
|  | Hs.395482 |
|  | Mm.254494 |

---

|  |  |
| --- | --- |
| Homologene ID | 7314 |

---

|  |  |
| --- | --- |
| Shape | Sickle-vertex |

---

|  |  |
| --- | --- |
| IPI ID | IPI00875543 |
|  | IPI00895396 |
|  | IPI00338858 |
|  | IPI00982478 |
|  | IPI00476238 |
|  | IPI00216218 |
|  | IPI00216217 |
|  | IPI00973349 |
|  | IPI00895208 |
|  | IPI00625418 |
|  | IPI01011193 |
|  | IPI00113563 |
|  | IPI00230629 |
|  | IPI00895269 |

---
